# Trending Now: Using Social Media to Predict and Track Disease Outbreaks

**DOI:** 10.1289/ehp.120-a30

**Published:** 2012-01-01

**Authors:** Charles W. Schmidt

**Affiliations:** Charles W. Schmidt, MS, an award-winning science writer from Portland, ME, has written for *Discover Magazine*, *Science*, and *Nature Medicine*.


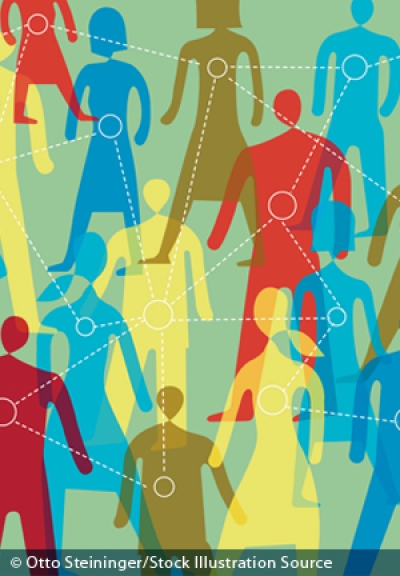
It’s winter, flu season, and you’re at your computer feeling a bit woozy, with an unwanted swelling in the back of your throat and a headache coming on. If you’re like millions of other people, you might engage in a moment of Internet-enabled self-diagnosis. You pop your symptoms into a search engine, and in the blink of an eye dozens of health-related websites appear on your screen. That search supplied you with information—some useful and some not—but in today’s hyper-connected world, it also supplied a data point for those who survey disease outbreaks by monitoring how people report symptoms via social media. In fact, social media, cell phones, and other communication modes have opened up a two-way street in health research, supplying not just a portal for delivering information to the public but also a channel by which people reveal their concerns, locations, and physical movements from one place to another.

That two-way street is transforming disease surveillance and the way that health officials respond to disasters and pandemics. It’s also raising hard questions about privacy and about how data streams generated by cell-phone and social-media use might be made available for health research. “There’s a challenge here in that some of these [data] systems are tightening in terms of access,” says John Brownstein, director of the computational epidemiology group at Children’s Hospital Boston and an associate professor of pediatrics at Harvard Medical School. “But we are seeing a movement towards data philanthropy in that companies are looking for ways to release data for health research without risking privacy. And at the same time, government officials and institutions at all levels see the data’s value and potential. To me, that’s very exciting.”

## Improving Surveillance

A pioneer in this field, Brownstein worked with collaborators at Children’s Hospital Boston to launch one of the earliest social media tools in infectious disease surveillance, a website called HealthMap (http://healthmap.org/) that mines news websites, government alerts, eyewitness accounts, and other data sources for outbreaks of various illnesses reported around the world. The site aggregates those cases on a global map, with outbreaks displayed in real time. Brownstein’s team recently launched Outbreaks Near Me, an iPhone application that delivers HealthMap directly to cell-phone users. Their newest endeavor is Flu Near You (https://flunearyou.org/), a website created with the American Public Health Association and the Skoll Global Threats Fund of San Francisco, California, which allows individuals to serve as potential disease sentinels by reporting their health status on a weekly basis.

Traditional flu surveillance by the Centers for Disease Control and Prevention (CDC) relies on outpatient reporting and virological test results supplied by laboratories nationwide. That system confirms outbreaks within about 2 weeks after they begin, but social media can flag more immediate concerns, according to Ashley Fowlkes, an epidemiologist in the CDC Influenza Division.

One of the CDC’s more recent collaborators is Google, to which millions of people turn for flu-related web searches. In September 2008, after the company’s researchers showed that spikes in flu queries and disease outbreaks often coincide,^^1^^ Google launched Google Flu Trends (http://www.google.org/flutrends/), a website that allows people to compare volumes of flu-related search activity against reported incidence rates for the illness displayed graphically on a map. According to Fowlkes, the CDC monitors Google Flu Trends as a potential source for early warnings in locations where health officials might want to mount a response.

But Fowlkes also cautions that online search behavior might have no bearing on whether an outbreak is really occurring. For instance, when the popular singer Rihanna announced (via Twitter) that she had the flu in October 2011, flu-related web queries surged. The timing of the spike and the search terms used (such as “Rihanna” alongside “flu”) suggest the queries were as much in response to public curiosity as anything else, Fowlkes says. “The Google Flu Trends system tries to account for that type of media bias by modeling search terms over time to see which ones remain stable,” she says. Otherwise, it would be vulnerable to “noisy” queries (i.e., those that might have nothing to do with changes in disease incidence).

Google doesn’t publicize its flu search terms for fear that malicious hackers might use them to undermine the system (for instance, by creating fake outbreaks). That’s unlike Twitter, a fast-growing “microblogging” platform used by hundreds of millions of registered users who collectively send more than 200 million “tweets” a day. Each tweet is at most 140 characters, which is limited but still long enough to add contextual information beyond what search terms can offer. That makes it easier to exclude noisy tweets, and it also allows scientists to mine for content describing what people think about treatments and other issues that could be crucial for delivering better outbreak responses, says Philip Polgreen, an associate professor at the University of Iowa Carver College of Medicine.

Scientists have found that tweet streams closely track reported cases of influenza-like illnesses (ILIs), conditions that cause fever with cough or sore throat but that aren’t necessarily influenza, which has its own viral etiology. In one study, Nello Cristianini, a professor at the University of Bristol, found that phrases containing terms such as “home worse,” “cough night,” “sore head,” and “swine flu” tracked with reported ILI outbreaks throughout the United Kingdom.^^2^^ And Polgreen found that terms including “flu,” “swine,” “influenza,” “symptom,” “shortage,” “hospital,” and “infection,” among many others, tracked user concerns during the H1N1 pandemic in 2009.^^3^^ What’s more, he reported, Twitter content predicted flu outbreaks 1–2 weeks ahead of the CDC’s surveillance average.^^3^^

Fowlkes emphasizes that although flu-related tweet streams correlate with the CDC’s ILI surveillance, they don’t always match up with laboratory-confirmed influenza.^^4^^ “All the social media systems need to be compared back with virologic data to see how well they correlate with true influenza infection,” she says. “Otherwise you risk treating the wrong people.”

Marcel Salathé, an assistant professor at The Pennsylvania State University, says that open access is in part what makes Twitter so promising as a health research tool. “I respect Google and what they’re doing with Google Flu Trends, but those data are closed and proprietary, so scientists can’t use them,” he says. “On the other hand, tweets are full of slang, but we can use machine-learning algorithms^^5^^ to make sense of those messages.”

## Mobile Insights

Meanwhile, Twitter’s growth pales with the meteoric rise of the cell phone. Among the world’s 7 billion people, an estimated 5.3 billion have cell-phone accounts, and cell-phone coverage extends to 90% of the global population.^6^ Every call made pinpoints the user’s location. Therefore, cell phones can track mass movements in which significant departures from the norm might reflect impacts from disease or migrations away from perceived threats. Scientists have long aspired to harness this capability to build elaborate models of social behavior. But advances have been slow in coming, in part because cell phone carriers—worried justifiably about compromising their user data—don’t supply ready access to that information.

Linus Bengtsson, a physician at the Karolinska Institute in Sweden, says that as it stands now, responders don’t have fast and accurate ways to track postdisaster migrations in developing countries. This has direct implications for health, he says, if it means responders don’t know where to find large groups of affected people. Cell-phone data could change that. In one of the few reports published on this topic so far, Bengtsson and colleagues showed that by mining anonymized cell-phone data it was possible to track population movements in Haiti after the 12 January 2010 earthquake and during the subsequent epidemic of cholera.^7^

For his research, Bengtsson petitioned Haiti’s largest cellular carrier, Digicel, headquartered in Kingston, Jamaica, for access to position data from roughly 1.9 million cell phones in the island nation corresponding to the period extending from 42 days before the earthquake until 158 days after. According to coauthor Johan von Schreeb, a surgeon from the Karolinska Institute, Digicel released the data only after company officials were satisfied that, as academics, Bengtsson and his team wouldn’t use them for economic gain.

Results from the study showed that an estimated 630,000 people who were in the Haitian capital of Port-au-Prince when the earthquake struck had left within 19 days. The major recipients of that outflow, the phone data revealed, were three coastal towns: Les Cayes on the southern coast, Leogane to the east of the capital, and Saint-Marc to the north. In addition, an estimated 120,000 people who were outside the capital prior to the quake moved into the city during the same 19-day period. These findings were consistent with a retrospective United Nations survey conducted 6 months after the disaster, the authors report. But, crucially, the movement patterns were far different than the Haitian government’s official real-time estimates, upon which relief operations relied.^7^

Bengtsson and colleagues subsequently used call records to investigate population movements after cholera took hold in Saint-Marc and surrounding areas. Their investigation revealed that many who left those areas wound up in Port-au-Prince and other urban centers to the north and south.^7^ “That’s important because we know that people who leave a cholera-affected area can carry the disease with them,” von Schreeb says.

Nathan Eagle, an adjunct assistant professor at the Harvard School of Public Health, proposes that changes in cell-phone use patterns also may reveal where outbreaks emerge in remote locations, the notion being that people sick with cholera and other diseases will move around less, altering their call patterns in ways that produce a distinct behavioral signature detectible with statistical algorithms. Eagle recently investigated that hypothesis in Rwanda after cholera took hold in August 2011. His unpublished findings generated an intriguing result: changes in call patterns did, in fact, occur, but they coincided with disruptive floods that predated outbreaks of cholera, a waterborne disease, by about 2 weeks.

Meanwhile, using call records to monitor outbreaks and population movements requires access to baseline data, without which changes in user patterns can’t be detected. Both Eagle and von Schreeb concur that obtaining this access poses major hurdles. Companies might be willing to form data-sharing partnerships with health agencies and nongovernmental organizations, von Schreeb suggests, assuming that uses are limited to research and charitable causes. In other cases, Eagle adds, companies might allow data access in exchange for analytical services focused on building better models for delivering service to cellular subscribers.

**How Accurate Are Social Media?**

Accuracy studies of how social media platforms cover epidemic health threats are rare. But one study, conducted by the consulting firm ICF Macro on behalf of the Agency for Toxic Substances and Disease Registry, found that blog and Facebook postings covering two environmental health issues—perchlorate in baby food and mold problems with drywall from China—matched up well with what appeared in official reports on the same topics. “In general, we found that what most people were saying was fairly accurate,” says Nicole Vincent, a senior associate with ICF Macro. The exception came with the “comments” sections that follow online coverage of these same topics by traditional media. “There’s a higher likelihood that people will pass around false information on these comments sections,” she says. “But then again, there’s going to be a lot more opinion and discussion about personal experiences [in these sections].” The research was presented at the American Public Health Association’s 2011 Annual Meeting.^11^

## Investigating Social Networks

Ideally scientists want as much individualized information as they can get to anchor social network predictive models in real-world data. The power of these models was illustrated in a 2010 study by two professors and long-time collaborators—Nicholas Christakis from Harvard University and James Fowler from the University of California, San Diego—who found that social network analyses can predict flu outbreaks earlier than traditional tracking methods.^8^

That finding arose from a study in which Christakis and Fowler randomly selected a group of Harvard University students, asked each to name a friend, and then compared flu incidence among the “friends” group to that among the nominating group. The underlying hypothesis—attributed to professor Scott Feld of Purdue University—is that when asked to name a friend, most people will refer to someone with a higher social standing (i.e., by naming a popular friend, the person makes him- or herself appear to be more popular). Given that they have more social contacts, individuals of higher social status—dubbed “central individuals” by Christakis and Fowler—have more opportunity to become infected with transmissible illnesses.

Sure enough, the researchers found that on average, the “friends” group got sick 13.9 days earlier than the nominating group. From that, they concluded that by identifying and monitoring central individuals in a given population, it might be possible to predict flu outbreaks in advance.^^8^^ Christakis and Fowler’s report of this study won the International Society of Environmental Epidemiology’s award for the best environmental epidemiology paper of 2010.^9^

Christakis and Fowler achieved these findings because their central individuals were clearly identifiable in a single defined population (i.e., the Harvard University student body). Whether the same approach would scale up in a larger regional, national, or even multinational context isn’t clear, according to Stephen Eubank, a professor at Virginia Polytechnic Institute and State University. That’s because when it comes to wide-scale transmission, what’s important may not be how many contacts an individual has within his or her own community, but whether that person’s social position puts them at the boundary of another adjoining group. “In that case, you only need two contacts,” Eubank explains, “one in community A and one in community B.”

As in physical contact networks, social position also matters in communication networks, Eubank says, in that people with perceived credibility and access to major followings can have a disproportionately large influence on public opinion. What’s more, contact and communication networks can interact with and influence each other, he says. For instance, when central figures in a communication network (e.g., health officials, TV anchors, and community leaders) raise alarms about a potential outbreak, the contact network shifts as people respond to that information.

Social media have made the communication network far more “democratic,” Eubank says, now that anyone with access to a computer and an Internet connection can transmit information worldwide. And that can have unpredictable consequences for the communication and contact networks alike, Eubank says. For intance, people might refuse vaccinations on the basis of something they’ve read about or heard online.

Josh Epstein, a professor at Johns Hopkins Medicine who collaborates with Eubank, uses a method called agent-based modeling to study emotional responses to information, particularly fear. He says some people will isolate themselves out of fear during an outbreak, with a “dampening” effect on transmission, while others might flee, with dangerous implications for the outbreak’s long-range spread. “Fear-inspired flight is a real phenomenon,” Epstein says. “And these days, infectious agents can really get around. Back in 2003, severe acute respiratory syndrome was on five continents within twenty-four hours.”

Both Eubank and Epstein work with a modeling consortium known as the Models of Infectious Disease Agent Study (MIDAS), to which the White House, the Department of Defense, and the Department of Health and Human Services have all turned for advice during large-scale disease outbreaks, including the 2009 H1N1 pandemic. Funded by the National Institute of General Medical Sciences in the National Institutes of Health, MIDAS has two missions, says its director, Jim Anderson: first, to advance the science of modeling infectious disease transmission, and second, to be ready in the event of a national emergency. For MIDAS, Eubank models “synthetic populations” grounded in transportation patterns, workplace demographics, and other societal data streams. By running simulations on those models, he can generate hypothetical figures with a high degree of centrality—he calls them “critical nodes”—and then calculate how disease transmission patterns change when those nodes are subtracted from the analysis.

“Policy makers can turn to the model and ask, ‘Based on what we know about airline schedules coming out of Europe, what’s the likelihood that an infected person will land in Chicago in [a particular] time window?’” Anderson says. “It’s like a war game played with infectious disease.” A typical goal, Eubank adds, is to find nonpharmaceutical methods for impeding an epidemic—for instance, by closing high-risk schools.

One crucial question is whether social media unnecessarily fans public fears or spreads misinformation that exacerbates pandemic threats. During an October 2011 World Health Organization conference on improving outbreak preparedness, director-general Margaret Chan was quoted saying that social media generate “background noise” and “rumors” that can challenge response efforts even as they make it “extremely hard for any country to hide a public health threat of international concern.”^^10^^

So, as they do in so many other scenarios, social media platforms present a double-edged sword for health tracking. As a portal for channeling the personal experience of billions of people, they are a true reflection of our society—the good, the bad, and everything in between. But harnessing the best those platforms have to offer could take the protection of public health to a new level.
